# The use of occlusive dressings: influence on excisional wound healing in animal model

**DOI:** 10.1590/acb371206

**Published:** 2023-01-13

**Authors:** Mariana Raquel Soares Guillen, Eline Lima Borges, Gilmara Lopes Amorim, Puebla Cassini Vieira, Antônio Carlos Martins Guedes, Luciola Silva Barcelos

**Affiliations:** 1 MSc. Universidade Federal de Minas Gerais – School of Nursing – Department of Basic Nursing – Belo Horizonte (MG), Brazil.; 2 PhD. Universidade Federal de Minas Gerais – School of Nursing – Department of Basic Nursing – Belo Horizonte (MG), Brazil.; 3 PhD. Universidade Federal Rural de Pernambuco – Department of Animal Morphology and Physiology – Pernambuco (PE), Brazil.; 4 PhD. Universidade Federal de Minas Gerais – School of Medicine – Medical Clinic Department – Belo Horizonte (MG), Brazil.; 5 PhD. Universidade Federal de Minas Gerais – Institute of Biological Sciences – Department of Physiology and Biophysics – Belo Horizonte (MG), Brazil.

**Keywords:** Wounds and Injuries, Wound Healing, Occlusive Dressings, Inflammation

## Abstract

**Purpose:**

To analyze the influence of occlusive dressing on the healing of excisional skin wounds in mice.

**Methods:**

Pre-clinical, comparative, and translational study. Mice were divided into three experimental groups: wounds occluded with hydrocolloid (HD) dressings, transparent polyurethane film (TF) dressings, and without occlusion (WO), monitored at three, six and 14 days, with eight animals each. Closure rate, infiltration of neutrophils and macrophages, measurement of tumor necrosis factor-α (TNF-α) and vascular endothelial growth factor (VEGF) and, histologically, angiogenesis were evaluated.

**Results:**

Wound closure was accelerated in the occlusive groups. There was a decrease in TNF-α levels in the HD group when compared to the WO and TF groups. Neutrophils accumulation decreased in the HD group. Increased dosages of macrophages were evidenced in the HD group, compared to the WO and TF groups. Levels of VEGF were increased in the TF and HD groups.

**Conclusions:**

It is suggested that the occlusion of wounds modulates the inflammatory response.

## Introduction

Complex wound is the more recently used term to group up those difficult healing wounds that are a public health problem in Brazil, contributing strongly to the distance from social life, negatively interfering with the quality of life and compromising the functional capacity of the subject. Therefore, there is an incessant search by health professionals, especially nurses, to understand the processes that can provide a brief wound repair, in order to support decision-making in clinical practice^1^^,^ 2.

Successful wound healing involves patient-centered care, bed preparation, and treating underlying causes. Among these precautions, the choice of the dressings stands out, which can be classified as passive, interactive and/or bioactive. The first is not considered favorable for use in complex lesions, being more suitable for covering the wound or as a secondary dressing. The latter is characterized by providing or stimulating the release of bioactive substances during the healing process. Interactive dressings are semi-occlusive or occlusive, provide a micro-environment considered ideal for healing, in addition to acting as a protective barrier against external aggressive agents^3^^,^ 4.

Occlusive dressings are available in countries, including Brazil, in different presentations, composition and structure, the most used being the hydrocolloid, hydrofiber, calcium alginate, charcoal, and foam types. Occlusive dressings reduce the loss of heat and fluids through the wound surface and help in autolytic debridement and the absorption of excess exudate, especially during the inflammatory phase of healing. In addition, they guarantee physiological hypoxia in the wound bed, maintaining an adequate environment for angiogenesis, resulting in shorter healing time and, consequently, a greater healing rate^5^.

Occlusive dressings are primarily recommended for the treatment of wounds, especially chronic wounds, that is, those that require longer and cost more to heal. Among the technologies available on the market, hydrocolloid is one of the most used types of dressing available in Brazilian health services. This is usually composed of sodium carboxymethylcellulose, pectin, and gelatin. The products vary in the thickness of the pads and in the structure of the outer layer, and, in general, the dressings are self-adhesive and impermeable to water^6^.

Although the hydrocolloid pad is routinely prescribed by health professionals, so far there have been no studies carried out and published in Brazil that investigate the molecular, morphological, and histological changes in the wound resulting from the action of this type of occlusive dressing.

The hypothesis is that controlled tissue hypoxia on the wound surface is fundamental for angiogenesis and, consequently, effective healing. The confirmation of the hypothesis with the adoption of the experimental animal model will allow the translation of the knowledge generated to the clinical practice, supporting healthcare providers in the choice of dressings for the treatment of wounds.

Therefore, the aim of this study was to analyze the influence of occlusive dressings on the healing of excisional skin wounds in C57BL/6 mice.

## Methods

This was a comparative, controlled, translational, and preclinical study. The procedures took place at the Angiogenesis and Stem Cell Laboratory of the Institute of Biological Sciences and at the Animal Experimentation Center of the School of Medicine, both at the Universidade Federal de Minas Gerais (UFMG).

The animals used were healthy, male C57BL/6 mice, aged 7 to 8 weeks old, weighing 17 to 20 grams. They were kept under temperature (24 °C) and light (12 h light/dark cycle) controlled conditions, in individual cages that were cleaned periodically and with free access to food and water.

All procedures were performed in accordance with the standards set out in the Guide for the Care and Use of Laboratory Animals (Institute of Laboratory Animal Resources, National Academy of Sciences, Bethesda, MD, United States of America, 1996), under conditions approved by the Institutional Animal Welfare Committee and Local Animal Ethics Committee (authorization number 117/15). The experimental procedures were carried out under aseptic conditions to avoid infections.

### Sample calculation

The sample size was calculated using a 95% confidence interval for the mean (significance level of 5%) and 80% chance of detecting the difference between the means (80% statistical power), with the number of animals per group [*N* = (4σ2 (Zcrit + Zpwr) 2) / D2], with reference to the mean and standard deviation of previous experiments: analyzed parameter - excisional wound; mean 1 (1,132.0); mean 2 (921.8); expected difference between means (18.57); standard deviation 1 (126.1); standard deviation 2 (115.9); standard deviation in % (11.9); standard deviation in decimals (0.12); significance criterion (95%); Zcrit (1.95); statistical power (85%); Zpwr (1.036), Zcrit+Zpwr ^2 (8.916); expected difference (0.19% in decimals); and *N* sample/group (7.27014), considering eight animals per group^7^.

The study consisted of three groups: intervention with hydrocolloid (HD) dressing, transparent polyurethane film (TF) dressing, and without occlusion (WO). Each group was monitored individually at three, six and 14 days after the origin of the excisional injury.

### Procedures and experimental design

The excisional skin wound model was used. The animals were anesthetized with xylazine 2% at a dose of 10 mg/kg and ketamine 10% at a dose of 100 mg/kg (manufacturer Syntec), prepared in sterile 0.9% saline and administered intraperitoneally. The animals were remained in a noiseless environment under a thermal blanket throughout the anesthetic recovery period. The trichotomy was performed with a portable device and antisepsis with 70% alcoholic solution of the animal’s dorsal region, with four excisional skin wounds made using a 5-mm diameter dermatological surgical punch, removing the entire extension of the skin tissue. The wounds were made with approximately 0.5 to 1 cm between each other. Subsequently, they were cleaned with 0.9% saline solution, and the procedures for applying the dressings were performed, according to the experimental design:

- Control group 1: WO (animals with the excisional skin wound model without occlusion);
- Control group 2: TF (animals with the excisional skin wound model occluded with transparent polyurethane film);
- Intervention group: HD (animals with the excisional skin wound model occluded with hydrocolloid dressing)^8^.

**Figure 1:**
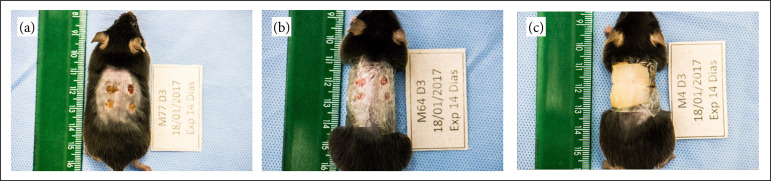
Animal of groups **(a)** without occlusion, **(b)** transparentpolyurethane film, and **(c)** hydrocolloid dressing (2017).

Mice were randomly divided into three groups (Fig. 1)–WO, TF, HD–, according to the experimental design (n = 24/group; totalizing 72 mice). Then, eight mice were allocated per analyzed time: three, six, and 14 days, and they were euthanized on the last day of analysis.

The dressing changes took place every 72 hours. This interval was defined as it is an adequate time for the dressing to remain on the animal’s back and it is an average interval for which the occlusive dressing remains on human wounds^9^.

Dressing changes were accompanied by procedures and evaluation performed with the animal under the effect of anesthetic, minimizing pain, discomfort, and stress during the manipulations. First, the anterior dressing was removed (TF and HD groups), then the wound was cleaned with 0.9% warm saline solution, in a single jet, with pressure obtained through the connection of a 25 × 8-mm needle to the 100-mL bag of 0.9% saline solution; the wound area was measured with a digital caliper, and the photographic record was made, with new dressings applied, according to the allocation of the groups. On days 3, 6 and 14, the wounds and the surrounding skin were also collected using an 8-mm punch.

At the end of each experimental period (days 3, 6 and 14), all the animals were euthanized with anesthetic overdose administered intraperitoneally, followed by cervical dislocation as recommended by the Animal Experimentation Ethics Committee that approved the study.

### Evaluations

#### Wound kinetics

The analysis of the kinetics was performed on days 0, 3, 6, 9, 12 and 14 by means of photographic record and measurement of the wound with a Dexter digital caliper, with measurement range from 0.01 to 150 mm. The results were expressed as a percentage of closure relative to the original wound size, using the following equation (Eq. 1):


Percentage of closure = (wound area) / (original wound area) × 100 (1)

The wounds of the 14^th^ day were photographed with a Samsung NX1 DSLR camera, 60-mm Samsung lens, 1:1 proportion, and analyzed by two evaluators regarding wound closure and scarring aesthetics. The judges received the photos of the animals from all groups (24 images) in the established segment and, in a masked way, classified the wounds according to the healing quality of the excisional wounds of the mice on the 14^th^ day: wound totally healed (aesthetically adequate, uniform, linear); wound partially healed (presence of flaking and/or crust, non-linear, aesthetically inadequate); or wound not healed.

### Quantification of neutrophil infiltration into the wound region

The indicator of neutrophilic infiltration in the wounds was evaluated through the activity of the myeloperoxidase (MPO) enzyme. Wounds and skin were frozen and stored at -20 °C until the day of tissue analysis. After thawing the tissue samples, they were weighed and homogenized in buffer (0.1M NaCl; 0.02M Na3PO4; 0.015M NaEDTA–pH 4.7) in the proportion of 1 mL of buffer for each 100 mg of tissue, using a tissue homogenizer (Ultra-turrax). Subsequently, the material was centrifuged at 10,000 g/10 minutes at 4 °C, and the precipitate was subjected to hypotonic lysis (1.5 mL of 0.2% NaCl solution followed by the addition of an equal volume of solution containing 1.6% NaCl and 5% glucose–30 s later).

After further centrifugation, the precipitate was resuspended in buffer 2 (0.05M Na3PO4; hexadecyl trimethyl ammonium bromide, Sigma 0.5% w/v–pH 5.4) in the proportion of 1 mL for each 100 mg of tissue. Subsequently, the homogenate was subjected to three freeze/thaw cycles using liquid nitrogen. The samples were again centrifuged at 10,000 g/15 minutes, and the supernatant was collected for the test. The samples were diluted in buffer 2 at the ratio 1:6^8^.

Myeloperoxidase activity was calculated by measuring changes in optical density (OD) at 450 nm using the reaction between tetramethylbenzidine diluted in dimethylsulfoxide (1.6mM) and H_2_O_2_ (0.5mM). The reaction was stopped with H_2_SO_4_, and the absorbance reading was performed on a spectrophotometer. The results obtained were expressed as a relative unit, according to the standard curve of the number of neutrophils *versus* OD that was obtained by processing purified neutrophils (> 95% purity) and used for the measurement of myeloperoxidase activity, a standardization previously performed in the laboratory. Results were expressed as change in OD per mg of wet tissue^10^^,^ 11.

### Quantification of macrophage infiltration into the wound region

The macrophage infiltration indicator was evaluated through the activity of the N-acetyl-β-D-glycosaminidase (NAG) enzyme. Wounds and skin were frozen and stored at -20 °C until the day of tissue analysis. After thawing the tissue, it was weighed and homogenized, using a tissue homogenizer (Ultra-turrax) with 0.9% saline solution (4 °C) containing 0.1% v/v of Triton X-100 (Merck) in proportion of 1.9 mL of solution for each 100 mg of tissue. Then, the sample was centrifuged at 4 °C 3,000 g/10 minutes. The supernatant was immediately collected and used for the NAG assay.

The reaction was started with the addition of 100 μL of p-nitrophenyl-N-acetyl-β-D-glycosaminidase (Sigma-Aldrich), dissolved in citrate/phosphate buffer (pH = 4.5), final concentration of 2.24 mM, to 100 μL of the sample (supernatant collected after centrifugation of the tissue) diluted in citrate/phosphate buffer (1:10). The reaction was carried out at 37 °C/10 minutes, in 96-well plates. The reaction was terminated by the addition of 100 μL of 0.2 M glycine buffer (pH = 10.6) and quantified in a spectrophotometer at 405 nm. Results were expressed as change in OD per mg of wet tissue.

MPO and NAG activities were used as neutrophil and macrophage accumulation indexes. These assays were expressed by OD obtained from the reading of the biochemical analyzer.

### Histological assessment

The collected samples went through the stages of dehydration, diaphanization, bathing, and paraffin embedding with 12 sequential baths, using a LUPTEC tissue processor (model PT05).

The paraffin blocks were subjected to microtomy with 5-μm thick sections. Then, the sections were stained with hematoxylin and eosin (HE) stain. They were analyzed under an optical microscope (40× objective, 10× eyepiece) and photographically recorded with a digital camera attached to the microscope, using the ImageProPlus software, version 4.5.0.29 for Windows 98/NT/2000. In all sections, the entire wound area was photographed.

In order to evidence angiogenesis, the number of capillaries in the wound region at moments D3 and D6 were counted. Histomorphometric analyses were performed by two independent evaluators, in a masked way. The capillary counting occurred in the first 20 fields photographed.

### Cytokine dosage

The cytokines evaluated were tumor necrosis factor-α (TNF-α) and vascular endothelial growth factor (VEGF), on the days 0, 3, 6 and 14. The evaluation was carried out through the enzyme-linked immunosorbent assay (ELISA) immunoenzymatic reaction, using antibodies acquired from the manufacturer R&D Systems (Minneapolis, United States of America)^8^.

### Statistical analysis

The data from the experiments were presented as mean ± standard error of the mean. The results were analyzed through analysis of variance (ANOVA) and the Newman-Keuls multiple comparison test. The two-way ANOVA test was used for the line graphs to verify the interaction between the independent variables time and group, followed by the Bonferroni post-test. Differences between groups were considered significant when *p* < 0.05.

The quantification and statistical analysis of the results were performed using the GraphPad Prisma, version 5.0 for Windows program (GraphPad Software, La Jolla, CA, United States of America).

The Kappa coefficient^12^ was used to assess the agreement between the evaluators regarding the qualitative classification of wound scarring. As an auxiliary analysis, the percentile bootstrap intervals were calculated with 95% confidence for the *k* coefficient, the results of which were analyzed based on the proposal by Landis and Koch^13^. The R (version 3.4.3) software was used in the analyses.

The interpretation of the Kappa scale coefficient, was considered as it follows:

- < 0.00: no agreement;
- 0.20: minimal agreement;
- 0.21–0.40: reasonable agreement;
- 0.41–0.60: moderate agreement;
- 0.61–0.80: substantial agreement;
- 0.81–1.00: perfect agreement.

## Results

The use of the dressings was tolerated by all animals in the study, with no animals excluded due to the presence of signs of infection. However, 13 animals out of the 16 in the 14-day segment, belonging to the TF and HD groups, had irritant dermatitis related to the use of the medical patches.

### Influence of occlusion on the temporal profile of closure of excisional skin wounds in mice

On the third day after the wound was made, there was a statistically significant increase in the percentage of wound closure of the animals in the TF and HD groups compared to the WO group (*p* < 0.001). There was also a significant difference between the TF and HD groups (*p* < 0.001).

**Figure 2:**
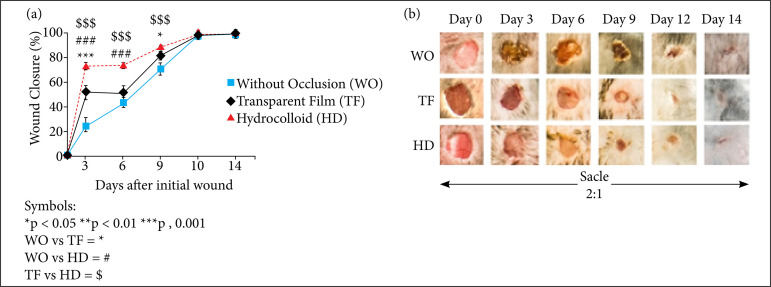
Influence of occlusion on the temporal profile of closure of excisional skin wounds in mice (2018). (a) Kinetics of wound closure in the animals of the WO, TF, HD groups. The wound closure rate results were expressed as a percentage of closure relative to the original wound size. The results were expressed as mean ± scanning electron microscope, *n* = 7-8 mice per group and time evaluated. (b) Representative photos of the evolution of the closure of excisional skin wounds in animals belonging to the WO, TF, HD groups. Belo Horizonte (MG), Brazil, 2018.

WO: without occlusion; TF: transparent film; HD: hydrocolloid; ***p < 0.001; *p < 0.05.

On the sixth day, the animals of the WO and TF groups presented wounds of similar sizes. However, when comparing the HD group with the other ones, there was a significant difference in the wound closure rate (*p* < 0.001), around 80% of closure, evidencing greater wound closure in the HD group compared to the other groups (Fig. 2).

On the nineth day, when comparing the percentage of reduction in the injured area, no significant difference was found between the WO and TF groups, or between the TF and HD groups. In this segment, there was a significant increase in the percentage of wound closure in the HD group when compared to the WO group (*p* < 0.05). After this period, there was equivalence between the groups regarding wound healing until the final day of the experimental evaluation.

### Influence of occlusion on the production of the TNF-α inflammatory mediator in excisional skin wounds in mice

**Figure 3:**
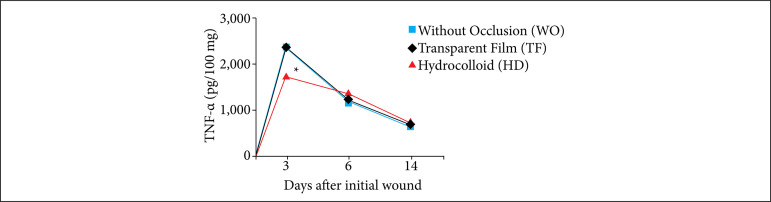
Influence of occlusion on the production of the TNF-α inflammatory mediator in excisional skin wounds in mice over the period of 0 to 14 days (2018). The concentration of TNF-α was significantly less on the third day in the group that used hydrocolloid dressings (in red) compared to the WO (in blue) and TF (in black) groups. The values represent the means (scanning electron microscope) of the groups of 7-8 animals per group).

WO: without occlusion–control group 1; TF: transparent film–control group 2; HD: hydrocolloid–intervention group; TNF-α: tumor necrosis factor-α; *p < 0.001.

The effect of the occlusion on TNF-α production resulted in a decrease in cytokine levels in the wounds of the group using the hydrocolloid dressings. This decrease was evident on the third day when compared to the WO and TF groups (*p* < 0.001). On days 6 and 14, there was no significant difference between the groups (Fig. 3).

### Influence of occlusion on the leukocyte profile in excisional skin wounds in mice.

**Figure 4:**
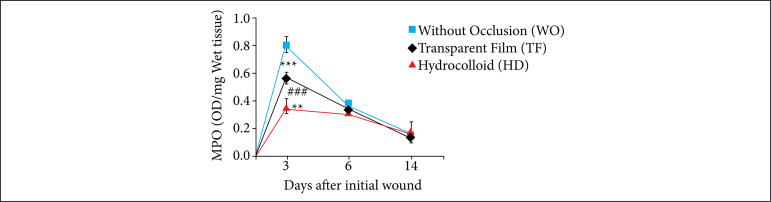
Influence of occlusion on the MPO activity in excisional skin wounds in mice (2018). MPO activity in skin wounds showed significant reduction on the third day in TF (in black) compared with WO (in blue) and HD (in red) groups. The values represent the means (scanning electron microscope) of the groups of 7-8 animals per group.

WO: without occlusion–control group 1; TF: transparent film–control group 2; HD: hydrocolloid–intervention group; MPO: myeloperoxidase; OD: optical density; ***p < 0.001 TF *vs.* WO group; ###*p* < 0.001 HD *vs.* WO and TF groups; **p < 0.01 TF *vs.* HD group (two-way analysis of variance).

The MPO activity was altered when the occlusion was performed with hydrocolloid dressings. This significantly decreased the MPO activity on the third day, when compared to the TF and WO groups (*p* < 0.001). Comparing the WO and TF groups, occlusion with TF significantly reduced MPO activity (***p < 0.001). Comparing with HD and TF groups, the former significantly decreased the MPO activity (**p < 0.01). On days 6 and 14, there was no significant difference in the between group assessments (Fig. 4).

**Figure 5:**
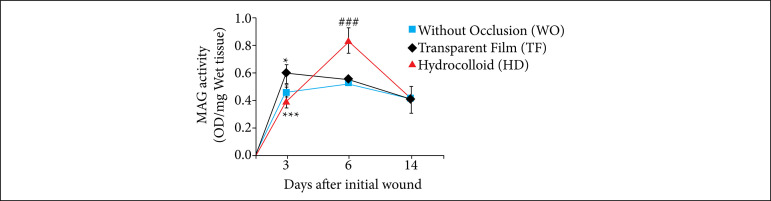
Influence of occlusion on the NAG activity in excisional skin wounds in mice (2018). NAG activity in skin wounds was significantly greater on the third day, in the TF group (*in black*) when compared to the WO group (*in blue*). Also on the third day, the HD group (in red) presented lower levels of NAG compared to the TF group (*in black*). On the sixth day, NAG levels were significant higher in the group occluded with HD, compared to the WO and TF groups. The values represent the means (scanning electron microscope) of the groups of 7-8 animals per group.

WO: without occlusion–control group 1; TF: transparent film–control group 2; HD: hydrocolloid–intervention group; OD: optical density; NAG: N-acetyl-β-D-glycosaminidase; *p < 0.05 TF *vs.* WO; ***p < 0.001 TF *vs.* HD; ###*p* < 0.001 HD *vs.* WO and TF groups (two-way analysis of variance).

The NAG activity levels were significantly higher on the third day in the TF group, compared to the WO group (*p < 0.05). At this moment, the HD group showed lower levels of NAG compared to the TF group (***p < 0.001). Day 6 was marked by significantly higher NAG levels in the hydrocolloid occluded group, in contrast to the groups with wounds without occlusion or occluded with transparent polyurethane film (***p < 0.001) (Fig. 5).

### Influence of occlusion on the angiogenesis of excisional skin wounds in mice

**Figure 6:**
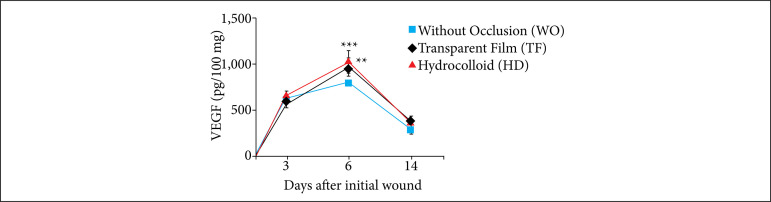
Influence of occlusion on VEGF in excisional skin wounds in mice (2018). Influence of occlusion on VEGFin skin wounds was significantly greater on the sixth day, in the TF (*in black*) *p* < 0.01 and HD (*in red*) groups compared to the WO group (*in blue*). The values represent the means (scanning electron microscope) of the groups of 7-8 animals per group.

WO: without occlusion–control group 1; TF: transparent film–control group 2; HD: hydrocolloid–intervention group; OD: optical density; VEGF: vascular endothelial growth factor; *p < 0.001.

The VEGF levels were significantly higher on the sixth day in the TF (**p < 0.01) and HD (***p < 0.001) animals, when compared to those of the WO group (Fig. 6).

### Influence of occlusion on capillary count

**Figure 7:**
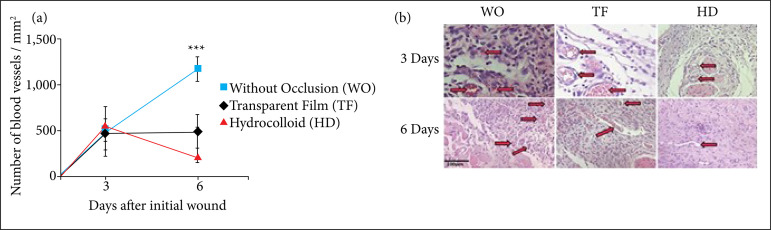
**(a)** Influence of occlusion on capillary count in excisional skin wounds in mice (2018). On the sixth day, an increase in the number of blood vessels/mm^2^ was observed in the skin wounds of the WO group (*in blue*), when compared to the TF (*in black*) (*p* < 0.001) and HD (*in red*) (*p* < 0.001) groups. The values represent the means (scanning electron microscope–SEM) of the groups of 6-7 animals per group. **(b)** Representative image of the influence of occlusion on capillary count in excisional skin wounds in mice (2018). Angiogenesis in the wound region. Representative photomicrographs of sections stained with hematoxylin and eosin (HE) three and six days after the wound was made showing the capillaries (*arrows*). Capillary density was assessed by blind counting in sections of tissue stained with HE, and it is represented as the number of vessels per mm^2^of granulation tissue. The results were expressed as mean ± SEM, *n* = 6-7 mice per group and time evaluated.

WO: without occlusion–control group 1; TF: transparent film–control group 2; HD: hydrocolloid–intervention group.

Regarding capillary count on the third day, there was no significant difference between the wounds with or without occlusion. However, the WO group showed significantly more vessels when compared to the TF (*p* < 0.001) and HD (*p* < 0.001) groups (Figs. 7a and 7b).

### The influence of occlusion on the qualitative analysis of the healing process of excisional skin wounds in mice

Kappa results for the classification of wounds by group and general, in which observations outside the main diagonal indicate disagreement between the evaluators (Table 1).

**Table 1:** Agreement analysis for the classification of wound healing. Belo Horizonte (MG), Brazil, 2018.

Group	E.1/ E.2	WTH	WPH	WNH	Kappa	95%CI*	*P*-value
HD (*n* = 8)	WTH	2	1	0	.53	[0.00–1.00]	0.048
	WPH	0	4	0			
	WNH	0	1	0			
TF (*n* = 8)	WTH	5	2	0	-.20	[-0.50–0.00]	0.537
	WPH	1	0	0			
	WNH	0	0	0			
WO (*n* = 8)	WTH	3	0	0	.75	[0.18–1.00]	0.029
	WPH	1	4	0			
	WNH	0	0	0			
General (*n* = 24)	WTH	10	3	0	.52	[0.19–0.83]	0.006
	WPH	2	8	0			
	WNH	0	1	0			

WO: without occlusion; TF: transparent film; HD: hydrocolloid; E.1: evaluator 1; E.2: evaluator 2; WTH: wound totally healed; WPH: wound partially healed; WNH: wound not healed;

* Bootstrap confidence interval; 95%CI: 95% confidence interval.

The evaluators disagreed on the classification of six wounds. There was moderate (*k* = 0.52 [0.19; 0.83]) and significant agreement between the evaluators (*p* = 0.006). In the HD group, the evaluators disagreed on the classification of two wounds. There was a moderate (*k* = 0.53 [0.00; 1.00]) and significant (*p* = 0.048) agreement between the evaluators. In the TF group, the evaluators disagreed on the classification of three wounds. According to the Kappa (*k* = 0-.20 [-0.50; 0.00]), the agreement between the evaluators was not significant (*p* = 0.537). In the WO group, there was disagreement on the classification of one wound. There was substantial (*k* = 0.75 [0.18; 1.00]) and significant (*p* = 0.29) agreement between the evaluators.

## Discussion

The use of occlusive dressings that remain on the wound for three to seven days or the daily change of dressings with ointments are conducts that raise doubts in practice. Thus, animal experimentation contributes to the understanding of knowledge gaps related to the physiological and pathological processes of wound healing^14^. Similar to the research presented, a study was carried out on the reduction of the injured area in mice with the hydrocolloid dressing. The animals were divided into three groups, which used hydrocolloid dressings, latex dressings (glove type), and without occlusion. During the first week, wound contraction was significantly reduced with the use of occlusive materials^15^. In the present study, wound closure was accelerated with hydrocolloid dressings or transparent polyurethane film, especially on the third day. Unlike the aforementioned study, the reduction in the injured area remained significant for wounds with hydrocolloid dressing occlusion on days 6 and 9.

Wound occlusion with hydrocolloid influenced the kinetics and also the MPO activity. There was a significant reduction in MPO on the third day, when compared to the TF and WO groups. However, on days 6 and 14 there was no difference in the assessments between the groups. There is a consensus that neutrophils are directly involved in killing invading microorganisms. Neutrophils are rapidly attracted to the wound region, releasing proteolytic enzymes, and eliminating bacterial and necrotic debris^16^.

The data suggest that the reduced MPO activity caused by occlusion is related to the low potential for contamination by microorganisms in these wounds. As microorganisms invade the wound, they can produce metabolites that are destructive to tissue and encourage cells to migrate to the wound site. However, there is a possibility that the less contact of wounds with the environment means less chance of the presence of invading microorganisms and, consequently, attenuated neutrophilic activity^17^.

In the late 20th century, occlusive dressings were prepared to provide moisture to the wound and hence protect it. Occlusive dressings aided in collagen synthesis, faster re-epithelialization, causing hypoxia, lowering the pH of the wound bed, which leads to less infection. In this category the hydrocolloid dressing is^18^. The data corroborate the results of the study regarding the reduction of MPO, which may be due to the reduction of contamination in the use of occlusive dressings.

The adequate activation of macrophages is essential in the repair of wounds; the macrophages are essential in the process of cleaning apoptotic neutrophils and coordinate the early closure of the wound. In their absence, wound cleansing shows an accumulation of neutrophils and an increased level of pro-inflammatory cytokines^19^^,^ 20.

In the present study, in contrast to the reduced neutrophil infiltrate, the findings suggest that occlusion with hydrocolloid dressings results in a higher level of macrophages, showing a peak on the sixth day. Therefore, it can be conjectured that the macrophages, evidenced here, are activated by the alternative route. This was evidenced by the statistically significant presence of macrophages, through occlusion with hydrocolloid, and the accelerated closure of the skin wounds in mice with their wounds occluded with hydrocolloid dressings^21^.

Cytokines are essential in all stages of the healing process. The potent pro-inflammatory cytokine TNF-α amplifies neutrophil chemotaxis and stimulates macrophages and the growth factors required in angiogenesis^22^. The data from the present study suggest that hydrocolloid intervention partially inhibits the pro-inflammatory cytokine TNF-α, as its concentration was significantly reduced on the third day after the injury in the group that used the dressing.

Elevated TNF-α expression is associated with delayed wound recovery, and low levels in wounds with an accelerated tissue repair process^23^. Histological changes and some molecular signals involved have been elucidated in the semi-occlusion of wounds in rats. Other study demonstrated that wounds that received treatment with semi-occlusive dressings demonstrated levels of pro-inflammatory cytokines interleukin 1-alpha (IL-1α), and TNF-α decreased significantly after the third postoperative day compared to the non-occlusive wounds^24^.

In diabetic rats, the increase in apoptotic cells led to an increase in the pro-inflammatory cytokines TNF-α in the tissue bed and in the wound fluid concomitantly with the reduction of the anti-inflammatory cytokine interleukin 10 (IL-10), which would normally act to decrease the inflammatory response. Furthermore, TNF-α levels were increased in chronic wounds, with this increase being related to the increase in the expression of metalloproteinases, and the excessive degradation of the local extracellular matrix by these cells^19^.

Growth factors were found in wound fluids occluded with dressings and demonstrated an important link with the stimulation of endothelial cells^16^^,^ 25. The vascular endothelial growth factor (VEGF) is released by a variety of cells, including macrophages, and stimulates multiple components of the angiogenic cascade. This factor is regulated during the first days of healing, when capillary growth is at its maximum. Experimental data support the hypothesis that VEGF stimulates epithelialization and collagen deposition in a wound, that is, it stimulates wound healing^26^.

In this study, VEGF levels were significantly increased on day 6 in those animals with wounds occluded with TF or hydrocolloid dressings, compared to the group without occlusion. This fact may demonstrate the benefit of this factor in the repair of wounds, since the deprivation of VEGF, due to proteolytic factors, has been considered as an underlying cause of healing difficulty^27^.

Different results were obtained in a study that aimed to describe the effects of the 35k protein on the healing process. In the treatment group, wounds were made, and isolated 35k protein was applied, with transparent polyurethane film dressing, while in the control group, phosphate buffer solution was used. There was an increase in the healing rate in the mid-stage of tissue repair in the intervention group. Considering VEGF levels, there was no increase in this factor, showing that the levels of this mediator would not be involved in accelerating wound healing and in the angiogenesis process. Despite remaining stable, an increase in the number of new vessels was observed, even though there were no changes in the level of VEGF and hypoxia-inducible factor 1-alpha (HIF-1α). The increase in angiogenesis did not occur through the VEGF and HIF-1α pathways^28^.

In the present study, a macroscopic assessment of healing was performed, which proved to be of crucial importance for monitoring the skin repair. Two evaluators with experience in the treatment of wounds in humans evaluated the photos of the D14 moment. There was agreement between the judges in the evaluation of healing in which occlusion occurred with hydrocolloid dressings and in the control animals, without occlusion. In the present study, there were no qualitative aesthetic differences in the wound healing, occluded or not.

Regarding the animal model, the formation of scarring has been shown to be reduced in areas kept moist, when compared to dry wounds. In another research, the author investigated the role of the epidermal barrier in relation to hypertrophic scarring in a rabbit animal model. The comparison between the occluded wound, in relation to the control, demonstrated that the occlusion presented less cellularity and epithelial thickness, regardless of the occlusive agent used. In addition, the same authors stated that the theory behind occlusion is based on the restoration of the homeostasis of the hydrophilic epidermal barrier, with it preventing the loss of hydration of the layers below the epidermis, which suggests less exuberant healing^29^.

In humans, the most used dressing for the treatment of hypertrophic scars is the silicone dressing. However, one service has used hydrocolloid with positive results in patients with challenging scars: a large post-burn hypertrophic scar on the hand and arm of a young patient, with a growing keloid on the wrist and a progressive scar on the lower eyelid. It is noteworthy that larger clinical trials are needed to evaluate the use of hydrocolloid in the treatment of hypertrophic scars and keloids^30^.

The microenvironment of the wound is susceptible to several influencing factors, such as the dressing, the cells surrounding the wound, and the atmosphere that it is in contact with. Likewise, factors such as humidity, oxygen content, pH, microbial load, and temperature directly influence, each one in its own way, the wound repair. The importance is highlighted of evaluating and monitoring this microenvironment, in order to properly decide which treatment method should be used, aiming to optimize the wound environment so that healing occurs dynamically, efficiently and with the best possible quality, following the clinical guidelines^31^.

The present study raises some viable considerations, such as that the occlusion, mainly with hydrocolloid dressings, translates into a beneficial tissue repair. However, the positive impact analyzed in the VEGF did not necessarily culminate in a new formation of vessels. From this point of view, it can be speculated that the hypoxic stimulus for angiogenesis seems to be more important when it occurs through stimuli caused to the blood vessel network. It should be noticed that the mice in this study were healthy, therefore it appears that healing occurs through the control of inflammation, to the detriment of angiogenesis. VEGF is a classic pro-angiogenic factor, but there are several another angiogenic factors released in the wound. Studies have been demonstrated the role of neutrophil and their products as angiogenesis inductor^32^^-^ 35. In this work, it was observed that WO group showed an increase in the number of blood vessels, non-related with VEGF concentration in their wounds. On the other hand, WO group showed significant increased MPO activity in the wounds, suggesting that the increase in neutrophils and their products in the tissue, in part, may have contributed to the angiogenic process.

Nevertheless, further studies are needed to better understand the relationship between occlusive dressings, oxygen reduction in the wound bed and angiogenesis.

## Conclusion

Partial inhibition of the pro-inflammatory cytokine TNF-α reduced MPO activity, and higher levels of NAG suggest that occlusive dressings modulate the inflammatory response in wounds. Molecular angiogenesis has been linked to occlusive dressings due to high levels of VEGF. Still, there was no difference between film occlusion and no wound occlusion in terms of capillary count. Hydrocolloid occlusion showed an increase in the number of capillaries in healthy mice, making it an important proposal for further studies.
